# Consequences of Lockdown During COVID-19 Pandemic in Lifestyle and Emotional State of Children in Argentina

**DOI:** 10.3389/fped.2021.660033

**Published:** 2021-07-14

**Authors:** María Victoria Fasano, Marcela Padula, María Ángeles Azrak, Ana Julia Avico, Marisa Sala, María F. Andreoli

**Affiliations:** ^1^Instituto de Desarrollo e Investigaciones Pediátricas, Hospital de Niños de La Plata - Comisión de Investigaciones Científicas-Provincia de Buenos Aires, Buenos Aires, Argentina; ^2^Centro de Matemática La Plata, Facultad de Ciencias Exactas, Universidad Nacional de La Plata, Buenos Aires, Argentina; ^3^Consejo Nacional de Investigaciones Científicas y Técnicas, Buenos Aires, Argentina

**Keywords:** COVID-19, pandemic, lockdown, children, emotional state

## Abstract

The implications of the coronavirus disease (COVID-19) lockdown measurements and social isolation in children and their parents are still unknown. The aims of this study were to examine the impact of COVID-19 lockdown on emotional state, feelings and lifestyle of children and their parents, to explore the association between parental characteristics and child well-being and to examine whether the impact of lockdown depends on socio-economic status. Parents completed an online survey including data about socio-demographic information, parent and child feelings and lifestyle during lockdown. Logistic regression and correlation analysis were used to establish associations between variables. In total, 814 parents with children between 4 and 11 were included in the study. According to parents, 69.5% of the children showed changes in their emotional state, 55.3% altered their routine and 62.6% showed sleep disorders. Families with lower socio-economic status were more worried about health, shortage of food and household income (*p* < 0.01). Parent and children concern about food/essential items were highly associated [OR (CI 95%) 13.0 (6.81, 26.5), *p* < 0.01]. Adverse children's emotional state was associated with parental feeling of loneliness (*r* = 0.35) and inversely associated with keeping a routine (*r* = −0.11). Sleep changes were inversely associated with keeping a routine and having a balcony/garden (*r* = −0.53 and −0.16). We conclude that lockdown affected emotional state and lifestyle of children and parents, which were strongly related. Routine and positive parental attitude supported children's well-being. Economic issues were an important concern in families with lower socio-economic status. Our findings can help to promote child health during lockdown.

## Introduction

The health, social and economic implications of the coronavirus disease 2019 (COVID-19) pandemic are still difficult to estimate. To contain and mitigate the spread of COVID-19, in March 2020 the Argentinean Government decided for strong lockdown measurements such as the cessation of school programs for children who consequently needed to remain at home. Although some restrictions started to ease over time, by November 2020 most of the schools still remained closed (1).

COVID-19 pandemic and lockdown measurements led to social isolation that affected severely the mental health of the general population all over the world, causing an increase in mental distress (2), depression and anxiety through the lockdown (3–7), sometimes associated with changes in feelings and lifestyle that include reduced physical activity, unhealthy eating habits, inadequate sleep quality and feeling of loneliness (2, 4, 7, 8). Family lifestyle was also drastically affected: parents suffered psychological distress due to unstable financial circumstances, school closures, and suspended educational services (9, 10). Children and adolescents also started to experience adverse emotional responses (stress, worry, helplessness, social and risky behavioral problems, anxiety, and depression) (11–16) and changes in lifestyle such as sleeping problems, increased screen exposure, reduced physical activity and unhealthy eating habits (17–20).

The lockdown measurements affected household finances with stronger implications for families with children living in poverty and/or crowded housing conditions (21–23). In the first half of 2020 Argentina's poverty rate rose to 40.9%, as reported by the country's official INDEC statistics agency (24), underscoring the devastating impact of the pandemic on the country's population. The lockdown presumably affected to a different extent people from different socio-economic status, and precise estimation of such impact is extremely valuable to decide future Government measures to address the consequences of the unprecedented crisis.

As mentioned above, several studies reported the effects of pandemic lockdown in adults (3, 4, 8, 25) and children (18, 19, 26–29) mainly in Asia and Europe, to our knowledge only one of these studies was conducted in toddlers and pre-schoolers from Latin America (26). In addition, few studies focused on parent-child dyads (10, 21, 22, 30). Thus, this study aimed to examine the impact of COVID-19 pandemic lockdown on emotional state, feelings and lifestyle of children and their parents in Argentina, focusing on their emotions, emotional stability, worries, routine, sleep, and daily activities. Also, the study explored the association between parental feelings and worries and child well-being. Furthermore, the study examined whether the impact of social isolation during the pandemics depends on the socio-economic status of the family.

## Materials and Methods

### Sample Selection

Parents filled out an anonymous online survey, after reading the written consent form and explicitly agreeing to take part of the study. The survey was conducted from May 26th to June 17th 2020, targeting parents of children aged 4–11 years-old. This age range was chosen to include children receiving pre-school and primary education. In case of multiple children, parents were asked to report on one child only. All questions were answered by the parent. The survey was conducted using an online platform, accessible through any device with an Internet connection. The survey was disseminated through institutional and private social networks (Twitter, Facebook, and Instagram), and institutional mailing lists. This method of administration provides a sample whose population parameters cannot be controlled as it is the case for probabilistic sampling. Such strategy was effective for the research objectives, because it facilitated the wide dissemination of the survey during a period with territorial restrictions due to the pandemic. The final sample included 814 families because respondents with missing or implausible data (*n* = 302, e.g., child age out of range) were excluded from the analyses. Inclusion criteria: adult (>18 years old) mothers and fathers with children 4–11 years-old. Exclusion criteria: adults who did not have children or whose children were out of the age range.

### Survey

The survey was specifically built using Google Form by the Institute of Development and Paediatric Research (IDIP), La Plata's Children Hospital, Buenos Aires, Argentina. For this, scientific literature related to the impact of lockdown on emotional state, feelings and lifestyle was reviewed (3, 4, 8, 12, 18, 28) and questionnaires applied in previous studies were considered for creating our survey. The survey was first tested in a small number of parents who were asked whether the questions were clear. The survey (Supplementary Table 1) included 43 closed questions, for each a list of acceptable responses was provided. Questions were divided into three different sections: (1) parent and family socio-demographic data (age, educational level, hometown, employment, telework, public health assistance, social welfare benefits, number of rooms in the house, number of persons living in the house, having a balcony/garden in the house, presence of pets), (2) children's data, feelings and lifestyle during lockdown (gender, age, worries about COVID-19, feelings and worries during lockdown, emotional state, routine, time spent in different activities, sleep, virtual contact with family/friends), (3) parent's feelings and worries during lockdown (worries about COVID-19 and feelings and worries during lockdown).

### Statistical Analyses

Statistical analyses were performed using the R software version 3.6.0. Quantitative variables are presented as median (interquartile range, IQR) and categorical data are summarized as frequency counts and percentages. Chi-squared test or Fisher's exact test were used to test for associations between categorical variables. Pairwise comparisons between multiple groups were adjusted by the Benjamini and Hochberg (BH) method (31). Logistic regression models were used to estimate the odds ratio (OR) and 95% confidence interval (CI) between children's and parent's feelings and worries. Associations between children's emotional state, sleep and daily activities and parent's feelings and socio-demographic factors were assessed by polyserial or polychoric correlations according to the nature of the variables. To identify possible socioeconomic status (SES) subgroups, we conducted a cluster analysis on the educational levels of parents, social welfare benefits, public health assistance, number of rooms in the house and number of persons living in the household. All statistical tests were two tailed/bilateral, and the significance level was set at *p* < 0.01.

### Ethics Approval

The study was approved by the Institutional Committee for the Revision of Research Protocols (CIRPI) of the Institute of Development and Paediatric Research (IDIP), La Plata Children's Hospital, and conducted according to the Declaration of Helsinki guidelines and Argentinian legal provisions governing clinical research on humans. We obtained informed consent from the participants included in this study.

## Results

### Family Features and Clustering Based on the Socio-Economic Status (SES)

Respondents were between 21 and 56 years-old (median: 39), primarily college or university students/graduates (87.17%), employees (62.9%) that during lockdown were working part-time (42.8%) and from home (68.9%). More than 13% reported receiving social welfare benefits and 12.3% were assisted in the public health system. Home residences predominantly had 2–3 rooms and 88.8% housed between 3 and 5 people. Based on this, the median for the ratio between the number of persons living in the household and the number of rooms in the house was 1.5. Children were uniformly distributed by gender and age (Supplementary Table 2). To assess if the impact of the lockdown depended on SES, we generated a 2-group partitioning of the families by conducting a cluster analysis including the following categorical variables: (1) public health assistance, (2) employment, (3) education, (4) reception of social welfare benefits, and (5) ratio between the number of persons living in the household and the number of rooms in the house (above or below 1.5). As a result, 378 parents (46.4%) were attributed to a high SES cluster, which included parents with high educational levels (university), low reception of social welfare benefits, low use of public health assistance and a number of persons living in the household/number of rooms in the house ratio <1.5, while 436 parents (53.6%) were attributed to a low SES cluster.

### Impact of COVID-19 Pandemic Lockdown on Children

#### Emotional State

According to their parents, 69.5% of the children showed changes in their emotional state. More than half of the children had adverse consequences on their emotional state: 46.1% of the parents reported mood instability in their children, 4.1% reported a nervous or aggressive mood and 3.8% sadness or crying. On the other hand, 10.7% of the children were happy during lockdown. Also 4.8% of the parents reported another type of emotional change in their children. The percentage of children who were happier under lockdown was higher between 4 and 6 years-old (14.4%) than children between 9 and 11 (7.4%, *p* < 0.001) (Supplementary Figure 1). The percentage of children with no changes in their emotional state was higher between 9 and 11 (41%, *p* < 0.001). No differences were observed between the low and high SES clusters (*p* = 0.574) or between boys and girls (*p* = 0.039).

#### Feelings

Feelings of children during lockdown are shown in Table 1. According to the parent's opinion, 27% of the children were worried about getting/transmitting the COVID-19, older children (9–11 years old) being more worried than younger children. More than 16% were afraid to leave the house. Most of the children missed visiting their relatives (90.4%) and attending to school (64.6%), independently of their age but more often among girls than boys (93.6 vs. 87.2%, *p* = 0.002 and 71.2 vs. 57.3%, *p* < 0.001, respectively). Children mainly between 6 and 7 years-old missed practicing sports (75.9%, *p* < 0.001) and their friends (89.1%, *p* = 0.002), with no significant gender differences. Regarding SES, children belonging to families in the low cluster were more worried about food or money shortage than children in the high SES cluster.

**Table 1:** Children's feelings and lifestyle during lockdown [*n* (%)].

	**Total**	**4–6 years**	**7–8 years**	**9–11 years**	***p*-value**	**Low SES**	**High SES**	***p*-value**
Belief that COVID-19 is a very important issue
A lot/completely	617 (75.8)	234 (67.2)^a^	176 (80.0)^b^	207 (84.1)^b^	** <0.001**	318 (72.9)	299 (79.1)	0.050
Worry about getting COVID-19
A lot/completely	220 (27.0)	72 (20.7)^a^	70 (31.8)^b^	78 (31.7)^b^	**0.002**	121 (27.8)	99 (26.2)	0.636
Worry about his/her friends getting COVID-19
A lot/completely	267 (32.8)	87 (25.0)^a^	82 (37.3)^b^	98 (39.8)^b^	** <0.001**	152 (34.9)	115 (30.4)	0.203
Fear to leave the house
A lot/completely	136 (16.7)	44 (12.6)^a^	49 (22.3)^b^	43 (17.5)^a, b^	**0.010**	75 (17.2)	61 (16.1)	0.707
Worry about transmitting COVID-19 to someone else
A lot/completely	95 (11.7)	23 (6.6)^a^	33 (15.0)^b^	39 (15.9)^b^	** <0.001**	57 (13.1)	38 (10.1)	0.191
Worry about not having enough food
A lot/completely	50 (6.1)	13 (3.7%)	14 (6.4)	23 (9.3)	0.019	37 (8.5)	13 (3.4)	**0.003**
Worry about not having enough money
A lot/completely	81 (10.0)	30 (8.6)	20 (9.1)	31 (12.6)	0.253	60 (13.8)	21 (5.6)	** <0.001**
Missing friends
A lot/completely	676 (83.0)	271 (77.9)^a^	196 (89.1)^b^	209 (85.0)^a, b^	**0.002**	348 (79.8)	328 (86.8)	**0.009**
Missing practicing sports
A lot/completely	527 (64.7)	200 (57.5)^a^	167 (75.9%)^b^	160 (65.0)^a, b^	** <0.001**	269 (61.7)	258 (68.3)	0.056
Missing their family (outside the household)
A lot/completely	736 (90.4)	316 (90.8)	203 (92.3)	217 (88.2)	0.322	393 (90.1)	343 (90.7)	0.812
Missing going to school
A lot/completely	526 (64.6)	216 (62.1)	151 (68.6)	159 (64.6)	0.283	286 (65.6)	240 (63.5)	0.557
Similar routine as before COVID-19		
Not at all/a bit	450 (55.3)	187 (53.7)	121 (55)	142 (57.7)	0.626	247 (56.7)	203 (53.7)	0.437
Changes in his/her sleep
Without changes	304 (37.4)	156 (44.8)^a^	72 (32.7)^b^	76 (31)^b^	**0.001**	168 (38.5)	136 (36.1)	0.627
Wake up frequently	55 (6.8)	35 (10.1)^a^	12 (5.5)^a, b^	8 (3.3)^b^		32 (7.3)	23 (6.1)	
Sleeps during day	13 (1.6)	4 (1.1)	4 (1.8)	5 (2)		8 (1.8)	5 (1.3)	
Goes to bed later	441 (54.2)	153 (44)^a^	132 (60)^b^	156 (63.7)^b^		228 (52.3)	213 (56.5)	
Time spent per day in contact with friends/family		
Phoning		
Less than once a day	507 (62.3)	219 (62.9)	140 (63.6)	148 (60.2)	0.701	264 (60.6)	243 (64.3)	0.278
More than once a day	307 (37.7)	129 (37.1)	80 (36.4)	98 (39.8)		172 (39.4)	135 (35.7)	
Via WhatsApp		
Less than once a day	281 (34.5)	131 (37.6)	85 (38.6)	65 (26.4)	**0.005**	157 (36)	124 (32.8)	0.375
More than once a day	533 (65.5)	217 (62.4)^a^	135 (61.4)^a^	181 (73.6)^b^		279 (64)	254 (67.2)	
Social media		
Less than once a day	552 (67.8)	261 (75)^a^	163 (74.1)^a^	128 (52)^b^	**0.001**	312 (71.6)	240 (63.5)	0.084
Once a day	82 (10.1)	31 (8.9)	30 (13.6)	21 (8.5)		43 (9.9)	39 (10.3)	
A few times a day	65 (8)	26 (7.5)^a^	15 (6.8)^a^	24 (9.8)^b^		28 (6.4)	37 (9.8)	
On and off throughout the day	71 (8.7)	16 (4.6)^a^	9 (4.1)^a^	46 (18.7)^b^		35 (8)	36 (9.5)	
Constantly	44 (5.4)	14 (4)^a^	3 (1.4)^a^	27 (11)^b^		18 (4.1)	26 (6.9)	
Online games		
Less than once a day	504 (61.9)	259 (74.4)^a^	135 (61.4)^a^	110 (44.7)^b^	**0.001**	269 (61.7)	235 (62.2)	0.555
Once a day	84 (10.3)	21 (6)	35 (15.9)	28 (11.4)		43 (9.9)	41 (10.8)	
A few times a day	67 (8.2)	29 (8.3)^a^	18 (8.2)^a^	20 (8.1)^b^		33 (7.6)	34 (9)	
On and off throughout the day	111 (13.6)	31 (8.9)^a^	24 (10.9)^a^	56 (22.8)^b^		67 (15.4)	44 (11.6)	
Constantly	48 (5.9)	8 (2.3)^a^	8 (3.6)^a^	32 (13)^b^		24 (5.5)	24 (6.3)	

*Data are presented as frequency counts and percentages. Values in bold indicate statistically significant difference (p <0.01). ^a, b^Post-hoc comparisons using BH method, different letters indicate significant differences at level 0.01 between age groups*.

#### Lifestyle Changes

As shown in Table 1, 55.3% of the parents reported that their children altered their routine during lockdown, independently of their age. Around 62% showed sleep disorders, mainly going to sleep at late hours, and this percentage increased with age. Girls showed more sleep disorders than boys (67 vs. 56.7%, *p* = 0.004). Most of the children communicated with their friends/family outside of the household at least once a day via WhatsApp (65.5%), social media (32.2%), or online gaming (38.1%), and this percentage increased with age. Social media was used by 14.1% of the children and 19.5% played online games constantly or on-and-off throughout the day, especially boys (*p* < 0.001 vs. girls). SES did not affect routine, sleep or virtual contact with friends/family.

#### Daily Activities

As shown in Table 2, 31.8% of the children spent <30 min/day being outside, and 36.2% spent <30 min doing physical activity (inside or outside), without gender differences. Concerning indoor activities, 57.7% of the children spent 2 h or more playing inside and 36.2% spent <30 min doing handicrafts. Most of the children (62.4%) spent <30 min a day reading. Regarding screen time, 28.1% spent more than 2 h playing screen games and 33.9% spent more than 2 h watching videos and/or TV. Also 21.6% spent more than 2 h playing screen games plus more than 2 h watching videos and/or TV.

**Table 2:** Time spent in different activities during lockdown [*n* (%)].

	**Total**	**4–6 years**	**7–8 years**	**9–11 years**	***p*-value**	**Low SES**	**High SES**	***p*-value**
Time spent outside
Less than 30 min	259 (31.8)	91 (26.1)^a^	70 (31.8)^a, b^	98 (39.8)^b^	**0.002**	136 (31.2)	123 (32.5)	0.706
Physical activity (outside or inside)
Less than 30 min	295 (36.2)	93 (26.7)^a^	78 (35.5)^a^	124 (50.4)^b^	** <0.001**	156 (35.8)	139 (36.8)	0.771
Playing outside
Less than 30 min	324 (39.8)	106 (30.5)^a^	88 (40)^a, b^	130 (52.8)^b^	** <0.001**	173 (39.7)	151 (39.9)	0.943
Playing inside
2 h or more	470 (57.7)	243 (69.8)^a^	132 (60)^a^	95 (38.6)^b^	** <0.001**	252 (57.8)	227 (60.1)	0.999
Time spent doing school homework
Less than 2 h	358 (44.0)	203 (58.3)^a^	83 (37.7)^b^	72 (29.3)^b^	** <0.001**	198 (45.4)	160 (42.3)	0.081
2–4 h	292 (35.9)	108 (31)	88 (40.0)	96 (39)		163 (37.4)	129 (34.1)	
5 or more hours	164 (20.1)	37 (10.6)^a^	49 (22.3)^b^	78 (31.7)^b^		75 (17.2)	89 (23.5)	
Doing craft/hand activities
Less than 30 min	295 (36.2)	79 (22.7)^a^	84 (38.2)^b^	132 (53.7)^c^	** <0.001**	155 (35.6)	140 (37)	0.662
Reading (alone or with someone)
Less than 30 min	508 (62.4)	198 (56.9)^a^	136 (61.8)^a, b^	174 (70.7)^b^	**0.003**	255 (58.5)	253 (66.9)	0.014
Playing (video) games with cell phone, tablet or computer
2 h or more	229 (28.1)	68 (19.5)^a^	51 (23.2)^a^	110 (44.7)^b^	** <0.001**	118 (27.1)	111 (29.4)	0.482
Watching videos/movies/cartoons on a screen (cell phone, tablet, or TV)	
2 h or more	276 (33.9)	112 (32.2)	68 (30.9)	96 (39.0)	0.125	144 (33.0)	132 (34.9)	0.603

*Data are presented as frequency counts and percentages. Values in bold indicate statistically significant difference (p <0.01). ^a, b, c^Post-hoc comparisons using BH method, different letters indicate significant differences at level 0.01 between age groups*.

Children spent time in different activities depending on their age and gender. Younger children (4–6 years-old) spent less time doing school homework than older children. Besides, younger children spent more time outside, doing physical activity, playing, doing handicrafts and reading than older children (9–11 years-old). On the other hand, older children spent more time playing screen games. Significant gender differences were observed in screen games and handicrafts: boys spent more time playing screen games (36.1% spent more than 2 h/day vs. 22.2% in girls, *p* < 0.001) and girls spent more time doing handicrafts (73.3% spent more than 30 min/day vs. 53.3% in boys, *p* < 0.001). Time spent in different activities was not affected by SES.

### Impact of COVID-19 Pandemic Lockdown on Families and Association to Children's Lifestyle and Emotional State

#### Parent's Feelings

Almost half (47.1%) of the parents were worried about getting/transmitting COVID-19 and 27.9% were afraid to leave the house for essential activities such as work or essential shopping. Besides, 59.1% reported being worried about their children's use of screen, and 68.4% found it stressful to keep children entertained during lockdown. Also, 16.6% of the parents felt lonely, 18.8% did not feel capable to help their child with school homework and 45.1% did not have time to play with their children. These worries and feelings were not affected by SES. In contrast, preoccupations about health (physical or mental), shortage of food/essential items, total household income and children's future were higher in families in the low SES cluster (Supplementary Table 3).

#### Associations Between Child and Parent Feelings and Worries During Lockdown

As shown in Table 3, parental fright to leave the house and concern about accessibility to food/essential items, household income and children's future were highly associated with similar worries in the children. Particularly, children whose parents were concerned about having enough food were more likely to be worried about food shortage during lockdown [OR (CI 95%) 13.0 (6.81, 26.5)].

**Table 3:** Odds ratio (CI 95%) between children's and parent's feelings and worries.

	**Child afraid to leave the house**	**Child worried about not having enough food**	**Child worried about not having enough money**
Parental fear to leave the house	**4.10 (2.80, 6.02)**	**4.73 (2.63, 8.70)**	**2.54 (1.59, 4.06)**
Parental concern about having enough food or essential items	**2.37 (1.59, 3.51)**	**13.0 (6.81, 26.5)**	**6.95 (4.31, 11.4)**
Parental concern about total household income	**1.99 (1.37, 2.89)**	**7.08 (3.55, 15.7)**	**6.23 (3.66, 11.2)**
Parental concern about children's future	**2.44 (1.65, 3.67)**	**5.03 (2.46, 11.7)**	**4.83 (2.75, 9.08)**

*Values in bold indicate statistical significance (p <0.01)*.

#### Associations Between Children's Emotional State, Sleep and Activities and Routine, Parent's Feelings, and Socio-Demographic Factors

Only selected associations (|*r*|>0.1, *p* < 0.01) (32) were highlighted here. As shown in Table 4, parental age and part-time working were positively associated with time spent doing school homework. The presence of balcony/garden was inversely associated with changes in child's sleep and positively associated with the amount of time spent outside. Keeping a routine similar to how things were before COVID-19 was inversely associated with adverse emotional state and with changes in sleep, and positively associated with the time spent doing school homework. The feeling of loneliness of parents was associated with adverse emotional state and sleep changes in children, while the feeling of being able to help with school homework was inversely associated with adverse emotional state. Having time to play with children was inversely associated with adverse emotional state and with changes in sleep. Time spent in physical activity, reading, playing videogames or watching a screen did not present association with routine, socio-demographic factors or parent's feelings (data not shown).

**Table 4:** Associations between children's emotional state, sleep and daily activities and routine, parent's feelings, and socio-demographic factors.

	**Adverse emotional state** ** (0 = No, 1 = Yes)**	**Changes in sleep** ** (0 = No, 1 = Yes)**	**Time spent outside**	**Time spent doing schoolwork**
Parent's age	−0.09	0.01	−0.02	**0.15**
Parent's work (full or part-time)	−0.03	−0.07	0.08	**0.11**
Having a balcony/garden (0 = No, 1 = Yes)	−0.07	**−0.16**	**0.30**	0.07
Routine similar to before COVID-19	**−0.11**	**−0.53**	0.08	**0.16**
Parent feeling lonely	**0.35**	**0.22**	−0.04	−0.07
Parent feeling capable to help child with schoolwork	**−0.32**	−0.04	0.02	0.10
Parent with time to play with their child	**−0.23**	**−0.16**	−0.01	−0.07

*Polyserial or polychoric correlations are shown, correlations in bold are statistically significant at level 0.01*.

## Discussion

To our knowledge, this is the first study investigating how COVID-19 pandemic lockdown affects lifestyle and emotional state of children and the links between child and parent well-being in the context of pandemic-associated lockdown in families from Latin America. Our study found that the socio-economic status of the family, the alteration of the routine as a consequence of the pandemic, the parental feelings and the access to a balcony/garden strongly affect children's emotional state and lifestyle.

Current results indicate that 69.5% of the parents reported changes in the emotional state of their children after 2 months of lockdown. Younger children showed more dramatic mood changes. The most frequent feature was mood instability. Feelings of worry, fear and longing for their relatives and friends and school were also frequently reported by most parents. Younger children were also happier to staying at home, which may reflect their interest in spending more time with their parents/caregivers. No gender differences were observed, in agreement with other studies in children (20, 27), although some studies in adolescents show higher depression and anxiety levels in females (16). On the whole, our results are in line with the observations of other authors. In Italian children, a self-reported study (20) showed frequent mood swings in nearly half of the responders along with anxiety and depression symptoms. Other study in Spanish and Italian children reported that 85.7% of the parents perceived changes in their children's emotional state and behavior during lockdown, mainly difficulty concentrating and boredom (15). Studies in Chinese children show behavioral and emotional problems (17.6%) (29) and anxiety and depression rates between 18% (27) and 24% (12). Although our study did not assess depressive or anxiety symptoms, the observed changes in the emotional state could precede mental health decline and further evolve into such anxiety, depression, and posttraumatic stress symptoms.

Our study found important changes in the lifestyle of children, mainly loss of routine and changes in sleep. One of the most reported stressors of parents under pandemic lockdown was the disruption of children's routine (33), which can be detrimental because routines help children feel safe and contribute to healthy habits (33). In line with our results, other studies also reported changes in sleep. Sleep time increased in Canadian and Spanish children (17, 18), and behavioral health was impaired in American children (22). Other authors (20) also showed alterations in routine and sleep in Italian children aged 6 to 14 in a self-reported study conducted through video-calls. Communication with relatives/friends outside the household was mainly sustained on a digital level, which increases screen time, but may be beneficial in lockdown circumstances. Regarding daily activities, we observed commonly found gender and age-related differences (18): older children were less active than younger children and spent more time with screens, and boys spent more time playing screen games (34). Sedentary behaviors (<30 min of physical activity, more than 2 h playing screen games and/or watching videos and/or TV) were observed in more than 35% of the children. The high rate of sedentary behaviors is in line with the above referred Spanish and Canadian studies (17, 18) that reported decreased physical activity and increased screen exposure during pandemic lockdown. Interestingly, a study performed in 2014 found that 24.5% of Argentinean children between 5 and 10 years-old did not meet the international requirements of physical activity and showed a sedentary behavior in front of screen (35). Therefore, the proportion of sedentary children during the COVID-19 outbreak was increased in Argentina, exacerbated by a decline in outdoor time. Spending time outdoors has already been associated with more physical activity, less sedentary time and improved sleep (18). Thus, children should be encouraged to play and be active, engaging in activities compatible with lockdown measures, to minimize the negative consequences of lockdown.

The COVID-19 crisis has particularly affected vulnerable populations, including families with young children, who face dual caregiver and/or breadwinner demands (21) in a context of increasing poverty rate in Argentina. Although emotional state, lifestyle and activities of the children during lockdown did not depend on the SES, parents with lower SES were more worried about their health and economic issues (income, food/essential items), and these worries were also evident in their children. These findings indicate that 2 months of lockdown have an unfavorable impact on the emotional well-being mostly of vulnerable families, in line with reports from other authors (21).

Our study also aimed to identify factors that helped to support children's well-being. The key features for children's well-being unmasked by the current study were keeping a routine, a positive attitude from the parents and having a balcony/garden. The latter favors outdoor time and sleep, but does not increase physical activity. Keeping a routine similar to how things were before COVID-19 improves sleep, emotional state and dedication to school homework. These results agree with other authors who reported that mood state is more strongly related to life changes than specific COVID worries (36). Being an older parent and part-time working also favor dedication to school homework, and parents with positive attitudes such as playing with their children or helping them with school homework have a favorable impact on their emotional state. On the other hand, parents feeling lonely negatively affect the child's emotional state and sleep quality, and parents who feel worried or afraid highly condition the children's fears and worries, especially about shortage of food and money. Other authors reported that the parents' difficulty to deal with lockdown is associated with parent's stress, which impacts on children's behavioral and emotional problems (30), and distress levels are also mediated by child's behavioral and emotional difficulties (10). This is in line with our findings that parents feel stressed to keep children entertained and do not find time to play with them, though spending a lot of time in the house. Our results and results from other authors (21, 30) highlight the strong links between parental psychological well-being and the well-being of their children. When children do not have a predictable routine and do not have emotional support from their parents, they may show distress evidenced by emotional and behavioral problems.

It was recently reported that school closure due to COVID-19 has adverse consequences on children's physical and mental well-being (37), and similar disruptions are evident in our study. School closure isolates and socially deprives children from contact with their peers and teachers and is an important element in routine changes. School closure also plays a key role in the increase of sedentary behaviors since schools, and particularly physical education classes, provide an adequate environment to promote active behaviors among children and adolescents (17). Finally, parents are left alone dealing with children's education and having children at home 24 h/7 d, while also have to manage home-working and childcare (30). Therefore, the relevance of school closure on children's well-being should be taken into account when adopting preventive COVID-19 measures.

One of the strengths of our study lies in the fact that it was conducted 2 months after the beginning of the lockdown measurements, a very critical moment of the pandemic in Argentina. However, some features of the present study should be considered. First, this is a cross-sectional correlational study, therefore we cannot reach a conclusion about the long-term impact of lockdown or determine a causal relationship between the variables studied, a longitudinal analysis of the effects of lockdown on children and their parents over time would help to better understand the long-lasting consequences of lockdown. Moreover, the answers of the survey were exclusively provided by the parents. This data collection method may provide less information than child reports or direct evaluation by experts. However, it should be kept in mind that self-reports are not adequate for young children and pandemic restrictions limits direct evaluation by experts. Despite these limitations, this study is the first to provide data on the repercussions of COVID-19 lockdown on Argentinean children.

In conclusion, current results show that 2 months of pandemic lockdown in Argentina affected emotional state and lifestyle of children and their parents. During the COVID-19 crisis, strong links between parental psychological status and the well-being of children were observed. Lockdown especially affected the emotional well-being of more vulnerable families. Although the impact of the pandemic lockdown seems inevitable, our results show the importance of keeping a consistent routine during school closure, with enough opportunities to play, read, rest, and engage in physical activity, trying to avoid spending too much time in front of a screen. Besides, support for parents who are facing a stressful experience should also be provided. Our findings can guide efforts to preserve and promote child well-being during lockdown, helping governments to decide the confinement rules to apply to children, especially regarding school closing. Confinement rules should be accompanied by recommendations and guidelines for parents and caregivers to help children (and adults) to cope with the COVID-19 crisis.

## Data Availability Statement

The raw data supporting the conclusions of this article will be made available by the authors, without undue reservation.

## Ethics Statement

The studies involving human participants were reviewed and approved by Institutional Committee for the Revision of Research Protocols (CIRPI) of the Institute of Development and Paediatric Research (IDIP), La Plata Children's Hospital. The patients/participants provided their written informed consent to participate in this study.

## Author Contributions

MFA, MF, and MP designed the study and wrote the manuscript. MF, MP, MÁA, AA, MS, and MFA collected, analyzed, and interpreted the data. MF performed the statistical analysis. All authors approved the submitted versions.

## Conflict of Interest

The authors declare that the research was conducted in the absence of any commercial or financial relationships that could be construed as a potential conflict of interest.

## Supplementary Material

Supplementary Figure 1 Emotional state of children during lockdown. Percentage of children displaying different emotional states during lockdown according to parental report.

Supplementary Table 1 Survey.

Supplementary Table 2 Description of parent and child characteristics (*N* = 814).

Supplementary Table 3 Parent's feelings and worries during lockdown [n (%)].
