# Supplementary figures and images for: Consequences of Lockdown During COVID-19 Pandemic in Lifestyle and Emotional State of Children in Argentina
Source: Front Pediatr. 2021 Jul 14;9:660033. doi: 10.3389/fped.2021.660033 (PMC8316589; doi:10.3389/fped.2021.660033)

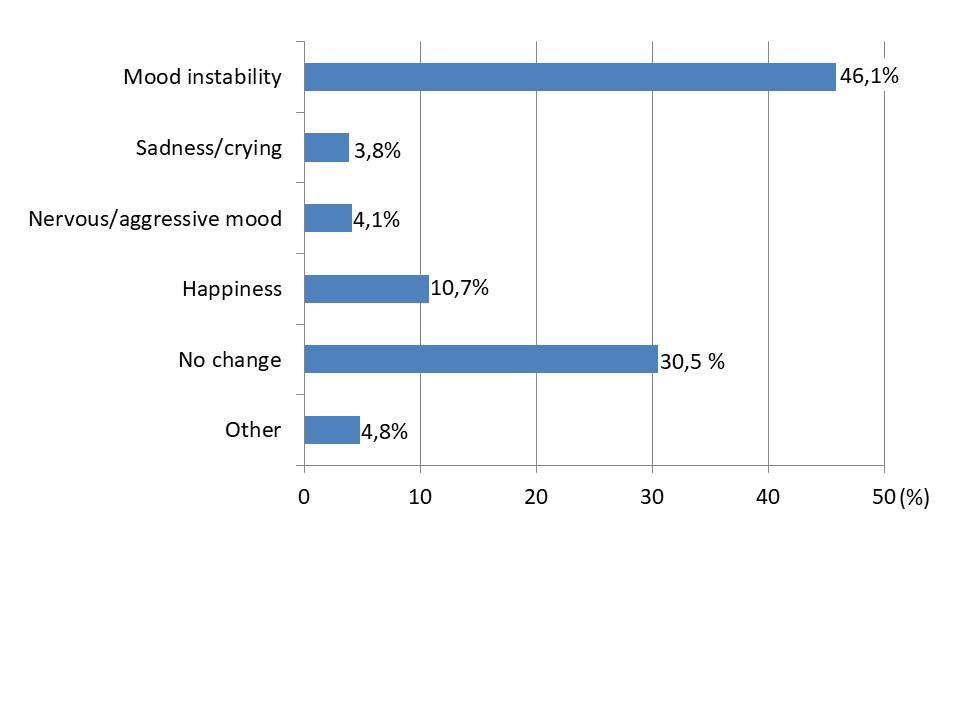

Supplement: Supplementary Figure 1 — Emotional state of children during lockdown. Percentage of children displaying different emotional states during lockdown according to parental report. [file Image_1.JPEG]
