# Supplementary material for: Consequences of Lockdown During COVID-19 Pandemic in Lifestyle and Emotional State of Children in Argentina
Source: Front Pediatr. 2021 Jul 14;9:660033. doi: 10.3389/fped.2021.660033 (PMC8316589; doi:10.3389/fped.2021.660033)
Supplement: Supplementary Table 1 — Survey. [file Table_1.pdf]

Suppl. Table 1: Survey

Questions	Answers
Parent and family socio-demographic data	
1. Age	Age in number
2. Educational level	High school or less
	College/University
3. Hometown	Province
4. Current employment	Housekeeper
	Employee
	Self employed
5. Work during lockdown	Full time
	Part-time
	Not working
6. Place of work during lockdown	From home/Out from the home (telework)
7. Use of public health assistance	Yes/No
8. Reception of social welfare benefits	Yes/No
9. Number of rooms in the house	Rooms in number
10. Number of persons living in the house	Persons in number
11. Presence of balcony/garden in the house	Yes/No
12. Presence of pets in the house	Yes/No
Children´s data, feelings and lifestyle during lockdown	
13. Gender	Female/Male/Prefer not to tell
14. Age	Age in number
Worries about COVID-19	
15. Does your child believe that COVID-19 is a very important issue?	Not at all/A bit/A lot/Completely
16. Is your child worried about getting COVID-19?	Not at all/A bit/A lot/Completely

17. Is your child worried that his/her friends get COVID-19?	Not at all/A bit/A lot/Completely
18. Is your child afraid to leave the house?	Not at all/A bit/A lot/Completely
19. Is your child worried about transmitting COVID-19 to someone else?	Not at all/A bit/A lot/Completely
<u>Feelings, worries and activities during lockdown</u>	
20. The emotional state of your child is…	the same as before the lockdown<br>happy<br>sad<br>nervous/aggressive<br>unstable mood<br>other
21. Is your child worried about not having enough food?	Not at all/A bit/A lot/Completely
22. Is your child worried about not having enough money?	Not at all/A bit/A lot/Completely
23. Does your child miss his/her friends?	Not at all/A bit/A lot/Completely
24. Does your child miss practising sports?	Not at all/A bit/A lot/Completely
25. Does your child miss his/her family who live outside the household?	Not at all/A bit/A lot/Completely
26. Does your child miss going to school?	Not at all/A bit/A lot/Completely
27. Does your child keep a similar routine as before COVID-19?	Not at all/A bit/A lot/Completely
28. Did your child show changes in his/her sleep?	Not at all/A bit/A lot/Completely
29. During the past week, how much time per day was your child in virtual contact with friends or family who do not live with you by telephone, via Whatsapp, using social media or using online games?	Less than once a day<br>Once a day<br>A few times a day<br>On and off throughout the day<br>Constantly

30. During the past week, how much time per day did your child spend in the following activities: being outside, doing physical activities, playing inside, doing school homework, doing craft/hand activities, reading, playing (video) games with cell phone, tablet or computer and watching videos/movies/cartoons on a screen (cell phone, tablet or TV)	Less than 30 min<br>30 min-2 hours<br>2-4 hours<br>5 hours or more

Parent´s feelings and worries during lockdown.

Worries about COVID-19

31. Are you worried about getting COVID-19?	Not at all/A bit/A lot/Completely
32. Are you worried about transmitting COVID-19 to someone else?	Not at all/A bit/A lot/Completely

Feelings and worries during lockdown

33. Are you afraid to leave the house?	Not at all/A bit/A lot/Completely
34. Are you worried about your children´s use of screen?	Not at all/A bit/A lot/Completely
35. Do you feel it is stressful to keep your child entertained?	Not at all/A bit/A lot/Completely
36. Do you feel lonely?	Not at all/A bit/A lot/Completely
37. Do you feel capable to help your children with their homework?	Not at all/A bit/A lot/Completely
38. Do you have time to play with your child?	Not at all/A bit/A lot/Completely
39. Are you worried about your physical health?	Not at all/A bit/A lot/Completely
40. Are you worried about your mental health?	Not at all/A bit/A lot/Completely
41. Are you worried about not having enough food or essential items?	Not at all/A bit/A lot/Completely
42. Are you worried about the household income?	Not at all/A bit/A lot/Completely
43. Are you worried about your children´s future?	Not at all/A bit/A lot/Completely

Suppl. Table 2 Description of parent and child characteristics (N=814)

Parent socio-demographic data	
Age (Median, IQR)	39 (36; 43)
Education, n (%)	
High School or less	105 (12.9%)
College/University	709 (87.1%)
Employment, n (%)	
Housekeeper	115 (14.1%)
Employee	512 (62.9%)
Self employed	187 (23%)
Work during lockdown, n (%)	
Full time	272 (33.4%)
Part-time	348 (42.8%)
Not working	194 (23.8%)
Place of work during lockdown, n (%)	
From home	464 (68.9%)
Out from the home (telework)	209(31.1%)
Public Health Assistance, n (%)	100 (12.3%)
Receives social welfare benefits, n (%)	108 (13.3%)
Number of rooms in the house (median, IQR)	2 (1;3)
Number of persons living in the house (median, IQR)	3 (2.3; 4)
Having a balcony/garden in the house, n(%)	775 (95.2%)
Presence of pets in the house, n(%)	586 (72%)
Children data	
Gender, n (%)	
Male	368 (45.2%)
Female	424 (52.1%)
Prefer not to tell	22 (2.7%)
Age	
Total (median, IQR)	7 (5; 9)
4-6, n (%)	348 (42.8%)
7-8, n (%)	220 (27%)
9-11, n (%)	246 (30.2%)

Suppl. Table 3 Parent´s feelings and worries during lockdown [n (%)].

	Total	Low SES	High SES	p-value
Worry about getting COVID-19				
A lot/completely	383 (47.1%)	209 (47.9%)	174 (46.0%)	0.622
Worry about transmitting COVID-19 to someone else				
A lot/completely	402 (49.4%)	221 (50.7%)	181 (47.9%)	0.440
Fear to leave the house				
A lot/completely	227 (27.9%)	133 (30.5%)	94 (24.9%)	0.085
Worry about children´s use of screen				
A lot/completely	481 (59.1%)	262 (60.1%)	219 (57.9%)	0.568
Feeling stressful to keep children entertained				
A lot/completely	557 (68.4%)	308 (70.6%)	249 (65.9%)	0.151
Feeling of loneliness				
A lot/completely	135 (16.6%)	85 19.5%)	50 (13.2%)	0.018
Feeling able to help children with homework				
A lot/completely	661 (81.2%)	346 (79.4%)	315 (83.3%)	0.151
Having time to play with my child				
A lot/completely	447 (54.9%)	228 (52.3%)	219 (57.9%)	0.120
Concern about physical health				
A lot/completely	338 (41.5%)	205 (47.0%)	133 (35.2%)	**<0.001**
Concern about mental health				
A lot/completely	395 (48.5%)	234 (53.7%)	161 (42.6%)	**0.002**
Worry about not having enough food or essential items				
A lot/completely	188 (23.1%)	124 (28.4%)	64 (16.9%)	**<0.001**
Worry about household income				
A lot/completely	340 (41.8%)	212 (48.6%)	128 (33.9%)	**<0.001**
Worry about children´s future				
A lot/completely	432 (53.1%)	253 (58.0%)	179 (47.4%)	**0.002**

Data are presented as frequency counts and percentages. Values in bold indicate statistically significant difference ($p<0.01$)
